# Current research of Assisted Reproductive Technology for women with early endometrial cancer and atypical endometrial hyperplasia after conservative treatment

**DOI:** 10.3389/fendo.2024.1377396

**Published:** 2024-06-11

**Authors:** Yan-le Jiang, Yan-ying Lin, Chen-xi Chen, Yu-xin Li, Huang-yan Xie, Bei-hong Zheng

**Affiliations:** ^1^College of Clinical Medicine for Obstetrics & Gynecology and Pediatrics, Fujian Medical University, Fuzhou, Fujian, China; ^2^Center for Reproductive Medicine, Fujian Maternity and Child Health Hospital, Fuzhou, Fujian, China

**Keywords:** assisted reproductive technology, endometrial cancer, atypical endometrial hyperplasia, fertility-sparing treatment, pregnancy outcome

## Abstract

As the incidence of endometrial cancer (EC) and atypical endometrial hyperplasia (AEH) has been increasing, and has shown young trend. It is crucial to study the fertility-preserving treatment of endometrial lesions and fertility-promoting protocols. Age, obesity, and irregular ovulation are not only high-risk factors for endometrial lesions but also key factors affecting female fertility. Assisted reproductive technology (ART) can significantly improve pregnancy outcomes in patients with AEH and EC after conservative treatment. Based on the existing studies, this article reviews the progress of research on pregnancy outcomes of ART and its influencing factors in such patients. It helps physicians in providing optimal fertility guidance.

## 1 Introduction

Endometrial cancer (EC) is one of the most common gynecologic malignancies (1), with the second highest incidence (2). In 2020, a total of 417,367 women were diagnosed with EC globally, which accounted for 4.5% of all malignancies in women (3). Atypical endometrial hyperplasia (AEH) is a precancerous lesion of EC. The risk of progression from atypical hyperplasia to endometrial cancer is estimated at 29% (4). In recent years, endometrial cancer has shown young trend. More than 5% of the diagnosed EC patients were aged between 35 and 44, and 2% were aged between 20 and 24 (5). To patients with the desire to have children, studying and proposing fertility-preserving treatment is important (6). The relevant guidelines of the European Society of Human Reproduction and Embryology stated that fertility-sparing treatment was considered for EC patients with Grade 1, Stage IA, and those without myometrial invasion or risk factors (7). A study has shown that fertility-preserving treatment for AEH and EC patients diagnosed with Grade 1, Stage IA was feasible and effective, achieving a complete disease remission rate of 84.5% and a pregnancy rate of 70.7% (8). The definite risk factors for endometrial cancer, such as obesity, polycystic ovary syndrome (PCOS), anovulatory cycles, and history of smoking (2, 9), are also factors that affect the natural conception of patients. Compared with patients without AEH/EC, patients with it have more reasons to choose ART. Using ART can shorten the time to the conception, thus avoiding prolonged, unopposed estrogen stimulation, which guarantees the reduction of disease relapse and progression. A study has shown that ART could improve the pregnancy and live birth rates in these patients (10). In this paper, we will review the research progress in pregnancy outcomes and its influencing factors in AEH/EC patients, to offer suggestions to practitioners for choosing the most suitable solution for such patients.

## 2 Material and methods

The literature search was conducted using CNKI, WANGFAN DATA, Web of Sciences, Yiigle and PubMed as electronic databases. Papers were identified by using a combination of the following text words: “fertility-preserving”, “endometrial cancer”, “atypical endometrial hyperplasia”, “assisted reproductive technology”, “frozen embryo transfer”, “*in vitro* fertilization”, “IVF-ET”, “pregnancy outcome”, “endometrial hyperplasia”, “live birth”, “conservative treatment”, “metabolic syndrome”, “hysteroscopic surgery”, “controlled ovarian stimulation”, “lynch syndrome”, “molecular classification” from 1985 to April 2024. A review of articles also included the abstracts of all references retrieved from the search. No restrictions for language or geographic location were applied. The eligibility of studies and the electronic search were independently assessed by two authors (Y.-L., J. and H.-Y., X.).

## 3 Pregnancy outcomes of ART treatment in AEH/EC patients after conservative treatment

For AEH/EC with reproductive needs, the goal of treatment is to maximize the rate of live births without increasing the rate of tumor recurrence. Several studies have shown that ART improved pregnancy and live birth rates in AEH/EC patients compared to natural conception or ovulation induction therapy (10–12). In addition, available studies suggested that pregnancy might be a factor in preventing the recurrence of endometrial lesions. A retrospective study published by Chae et al. (13) of stage IA, G1-G2 EC patients showed that the tumor recurrence rate was greatly lower in the pregnancy group than in the non-pregnant group. The time to recurrence was longer in the pregnancy group than in the non-pregnant group. A prospective cohort study by Maïlys Vaugon et al. (14) found that patients with AEH/EC who underwent IVF treatment had a higher clinical pregnancy rate and a lower rate of endometrial lesions recurrence compared to those who did not. In a prospective cohort study by Maïlys Vaugon et al. (14), the 2-year recurrence was found to be 20.5% in the pregnance while 62.0% in non-pregnance. The total recurrence rate of IVF group was 38.7%, while no-IVF group was 41.4%, with no statistical significance. Similarly, in another retrospectively analyzed study, the total recurrence rate of patients receiving ART was 22.2%, with no major differences in total recurrence rate between ART group and no-ART group. The 5-year recurrence rate in the group with live births was approximately 20%, while in the group without live births, the rate was approximately 60% (15). The results of the above data are promising, for the prolonged exposure to endogenous progesterone during pregnancy can reduce the recurrence rate of endometrioid cancer. Progesterone can antagonize the pro-proliferative effect of estrogen. It reduces the likelihood of estrogen-induced AEH, even EC. Although we have no clear evidence showing the different recurrence rates between ART group and no-ART group, it’s clear that ART can improve pregnancy outcomes for patients. Therefore, this group of patients should get pregnant as soon as possible after the remission of endometrial lesions, and the use of ART is recommended to speed up the pregnancy process. However, even though the recurrence rate is lower in pregnant women than in non-pregnant women, close follow-ups after pregnancy are still necessary.

On the other hand, although the effect of ART treatment is positive, adverse pregnancy outcomes and maternal and neonatal complications after ART still require attention. A retrospective cohort study (259 transplant cycles in AEH/EC patients) showed a clinical pregnancy miscarriage rate of 34.8% and a preterm birth rate of 16.7%. Obstetric risks such as hypertension during pregnancy, and gestational diabetes are increased, as well as neonatal risks such as low birth weight and macrosomia (16).

In conclusion, the pregnancy rate of ART-treated AEH/EC patients is higher than that of natural pregnancies, while the disease recurrence rate is lower. Therefore, for this group of patients, ART indications can be flexible, and at the same time it provides adequate pre-treatment counseling on the possible risks of ART treatment.

**Table 1** shows the comparison of factors influencing pregnancy outcomes in the studied patients.

**Table 1:** Comparison of factors influencing pregnancy outcomes in the studied patients.

Study Year	Type of Study	Pregnancy outcomes	Before ART							In the process of ART
			Pathological type(% or n)	Age(years)	BMI(kg/m^2^)	Comorbidity	Intrauterine environment	Conservative Treatment	Time to CR(months)	Conception protocol
He YJ, 2023 (8)	Retrospective	Pregnancy(% or n) Live birth(% or n)	overall 31.2% AEH 32.1% EC 29.7% overall 24.7% AEH 26.8% EC 21.6%	N/A	N/A	N/A	N/A	N/A	N/A	N/A
Xu Z, 2020 (10)	Retrospective	Pregnancy(% or n) Live birth(% or n)	CEH 50.0% EC 65.5% CEH 63.0% EC 68.4%	>35 61.9% ≤35 53.2% >35 46.2% ≤35 72.7%	>30 37.5% ≤30 62.7% >30 55.5% ≤30 67.6%	PCOS 50.0% IR 56.0% PCOS 50.0% IR 57.1%	N/A	LNG-IUS 46.7% MA 60.7% LNG-IUS + MA 60.0% LNG-IUS 64.5% MA 76.5% LNG-IUS + MA 53.3%	>6 52.0% ≤6 56.9% >6 61.5% ≤6 66.7%	ART 73.5% Natural 42.9% ART 84.0% Natural 42.9%
Xing Y, 2021 (12)	Retrospective	Pregnancy(% or n)	AEH 25% EC 62.5%	≥35 18.1% <35 43.4%	≥25 41.2% <25 36.0%	PCOS 0% IR 0% Hypertension 0%	IUA 1patient	N/A	3~12 0.33% 13~24 48.0%	ART 31.8% Natural 55.0%
Chae SH, 2019 (13)	Retrospective	Pregnancy(% or n)	EC G1 51.4% EC G2 18.2%	N/A	N/A	PCO on ultrasonography 45.5%	N/A	MPA 500 mg once daily 51.5% MPA 1000 mg once daily	N/A	N/A
Zong X, 2023 (16)	Retrospective	Pregnancy(% or n) Live birth(% or n)	N/A	N/A	N/A	N/A	N/A	N/A	N/A	Fresh ET 37.7% Frozen ET 31.0% Fresh ET 23.8% Frozen ET 17.8%
Morimoto C, 2022 (17)	Retrospective	Pregnancy(% or n) Live birth(% or n)	CAH 29.2% EC 26.4% CAH 30.3% EC 16.7%	N/A	N/A	N/A	N/A	N/A	N/A	N/A
Inoue O, 2016 (18)	Retrospective	Pregnancy(% or n)	AEH 40.5% EC G1/G2 49.2%	N/A	N/A	PCO on ultrasonography 55.2%	IUA 25%	N/A	N/A	Natural insemination 59.4% No infertility treatment 69.2% Timing treatment 50% Clomifene Citrate-timing treatment 57.1% HMG-timing treatment 53.8% IUI 37.5% IVF/ICSI 39.4%
Fan Y, 2021 (19)	Retrospective	Pregnancy(% or n)	AEH 59.0% EC G1 52.4% EC G2 25.0%	N/A	<24 85.7% ≥24 38.3%	Diabetes 72.7% IR 52.4% Hypertension 66.7% Thyroid diseases 55.6% PCO on ultrasonography 45.9% PCOS 51.9%	IUA 38.5%	Treatment protocol MPA 250 mg, once daily 53.3% MPA 500 mg, once daily 37.5% MA 160–320 mg, once daily 55.6% GnRH-a 60.0% Adjuvant metformin 40% Maintenance therapy None 45.5% Progestin 60.0% LNG-IUS 33.3%	< 6 70.0% ≥ 6 28.6%	Natural 36.8% Ovulation induction ± IUI 55.6% IVF-ET 60.0%
Du X-G, 2018 (20)	Retrospective	Pregnancy(% or n) Live birth(% or n)	N/A	N/A	N/A	N/A	N/A	N/A	N/A	One IVF cycle 51.7% GnRH-a 53.9%; Prolonged GnRH-a 75.0%; Long GnRH-a 75.0%; Short GnRH-a 50.0% Two IVF cycles 54.6% Letrozole 50.0%; Non letrozole 57.1% Three IVF cycles 42.9% Four and five IVF cycles 100% One IVF cycle 48.3% GnRH-a 53.9%; Prolonged GnRH-a 62.5%; Long GnRH-al 75.0%; Short GnRH-a 50.0% Two IVF cycles 31.8% Letrozole group 37.5%; Non letrozole group 28.6% Three IVF cycles 14.3% Four and five IVF cycles 0%
Xiao Z-R, 2020 (21)	Retrospective	Pregnancy(% or n)	AEH 55.9% EC 59.4%	≤35 66.7% >35 26.7%	≤30 56.0% >30 57.1%	Thyroid diseases 100% PCOS 69.2% Diabetes 37.5% IR 40.9% Hypertension 0%	IUA 20%	N/A	N/A	ART 59.5% Natural 54.2%
Guo Y, 2022 (22)	Retrospective	Live birth(% or n)	AEH 44.2% EC 32.1%	>35 22.9% ≤35 48.9%	<25 37.5% ≥25 44.8%	N/A	Thin endometrium 14.2%	N/A	N/A	Fresh ET 25.6% Frozen ET 20.4%
Chen J, 2021 (23)	Retrospective	Pregnancy(% or n)	N/A	N/A	N/A	N/A	N/A	N/A	N/A	PPOS 40.54% CC/LE+Gn 4.38% Standard regimen 21.43%

AEH, Atypical endometrial hyperplasia; EC, Endometrial cancer; CR, Complete remission; N/A, Not applicable; BMI, Body mass index; PCOS, Polycystic ovary syndrome; PCO, Polycystic ovary; IR, Insulin resistance; CEH, Complex endometrial hyperplasia; LNG-IUS, Levonorgestrel-releasing intrauterine system; MA, Megesterole acetate; ART, Assisted reproductive technology; IUA, Intrauterine adhesion; MPA, Medroxyprogesterone acetate; CAH, Complex atypical hyperplasia; IUI, Intrauterine insemination; IVF, In vitro fertilization; ET, Embryo transfer; IVF-ET, *In vitro* fertilization and embryo transfer; ICSI, Intracytoplasmic sperm injection; GnRH-a, Gonadotropin-releasing hormone agonist; PPOS, Progestin primed ovarian stimulation; CC, Clomiphene citrate; LE, Letrozol; Gn, Gonadotropin.

## 4 Factors affecting pregnancy outcomes of ART treatment in AEH/EC patients

### 4.1 Factors affecting pregnancy outcome: before ART

#### 4.1.1 Age and ovarian reserve function

The effect of age on female fertility is well known. Age plays a negative role in women over 35 years old, with a sharp decline in live births after age 40 (24). According to some studies, the pregnancy rate of ART treatment in AEH/EC patients gradually decreased with increasing age (12, 17–19). The prevalence of diminished ovarian reserve (DOR) is 1% to 3% in women of reproductive age (25). Du Xiaoguo et al. (20) found that the prevalence of DOR in AEH/EC patients was higher than that of normal women, suggesting that AEH/EC may negatively affect ovarian reserve function. Increasing the dose of gonadotropins (Gn) in AEH/EC patients will not increase the number of oocytes acquired, suggesting that the follicles in those patients are not as sensitive to exogenous Gn as normal ones (26). This suggests that the ovarian reserve function is somewhat affected.

#### 4.1.2 Menstrual disorders and polycystic ovary syndrome

Menstrual abnormalities like PCOS are more common in patients with AEH/EC, and PCOS is highly associated with endometrial lesions (2). One study showed that 41.6% of AEH/EC patients suffered from PCOS (20). PCOS leads to an endogenous hyperestrogenic state, which affects the remission and increase of recurrence of endometrial lesions (27). PCOS will result in elevating endogenous androgen and serum luteinizing hormone concentrations. They both have negative effects on fertility (28). PCOS patients are also prone to a combination of overweight and obesity, affecting oocyte quality (29). It leads to a reduction in overall fertility in this group of patients.

#### 4.1.3 Metabolic syndrome

Metabolic syndrome (MetS) includes a series of metabolic disorders such as insulin resistance (IR), hypertension, dyslipidemia, and so on (30). MetS is a definite risky factor for endometrial lesions (31). And MetS similarly affects female fertility. Hyperinsulinemia disrupts the ovarian microenvironment and reduces fertilization rates and the potential of embryonic development (32). Obesity can affect the hypothalamic-pituitary-ovarian axis, leading to sporadic ovulation or anovulation. It can also affect oocyte/embryo quality and the endometrial environment (33). In a retrospective study of 119 AEH/EC patients by Xing Yan et al. (12), none of the patients with comorbid diabetes and hypertension got pregnant. In a retrospective study of 107 AEH/EC patients by Xiao Zerui et al. (21), the pregnancy rate was 37.5% in patients with comorbid diabetes mellitus and 40.9% in patients with comorbid IR. The above data suggest a less favorable pregnancy rate in AEH/EC patients with combined MetS.

#### 4.1.4 Endometrial and intrauterine conditions

##### 4.1.4.1 AEH/EC classification staging

The grade of endometrial cancer has been recognized as an independent factor associated with pregnancy outcomes. A retrospective study showed that the endometrium of EC with Grade 1, Stage IA patients was more sensitive to progesterone compared to stage IA G2 patients (13). In a retrospective study by Yaxing Guo et al. (22), the live birth rate was higher in patients with AEH than in patients with EC. Chae et al. (13) found that the pregnancy rate of endometrial cancer was higher in the EC IA stage G1 group than in the EC IA stage G2 group. The above studies suggest that high-grade endometrial lesions may be an negative factor affecting reproductive outcomes.

In addition, the genetic factor influencing patients with AEH/EC cannot be ignored. Patients with Lynch syndrome (LS) or hereditary nonpolyposis colon cancer (HNPCC) are at high risk for EC (34). LS is a genetic disorder mostly associated with EC, which also has a negative impact on pregnancy outcomes. A study showed that LS patients positively responded to conservative treatment, but none of them achieved pregnancy, even had easy recurrence of disease (35). Alterations in some important oncogenes or anti-oncogenes, like PTEN, also have impact on fertility preservation. There are some genotypes of PTEN, among which PTENmut-Clin was an independent risky factor for unfavorable fertility-preserving treatment outcomes. However, no significant difference was found in the one-year cumulative pregnancy rates and cumulative live birth rates between PTENmut-Clin groups and PTEN-others groups (36). As for these patients, there is no sufficient evidence to show whether ART leads to a shorter time to pregnancy, increase fertility, or alteration of disease progression. Therefore, a personalized evaluation is necessary for each patient, and fertility treatment options should be individualized.

##### 4.1.4.2 Thickness of endometrium and whether combined with uterine adhesions

In the follow-up of endometrial lesions, we found repeated endometrial biopsies may cause irreversible mechanical damage to the endometrium. It leads to uterine adhesions and thin endometrium, which affects embryo implantation. In a retrospective study of 107 AEH/EC patients, Xiao Zerui et al. (21) showed that the clinical pregnancy rate in patients with uterine adhesions was lower than that in patients without it. Elizur et al. (37) found thinner endometrial thickness on hCG day in AEH/EC patients. In a retrospective study by Fujimoto et al. (38), none of the AEH/EC patients with endometrial thickness < 7 mm got live births, and the patients with AEH/EC had thinner endometrial thicknesses and lower rates of implantation compared with normal controls. In a retrospective study of 123 AEH/EC patients published by Yaxing Guo et al. (22), the proportion of thin endometrium in the non-live birth group was higher than that in the live birth group, suggesting that a thin endometrium was an independent risk factor affecting the outcome of live birth in AEH/EC patients. The above findings show that thin endometrium as well as uterine adhesions in those patients adversely affect pregnancy outcomes.

In addition, the intrauterine environment also affect pregnancy. A study showed that in 577 infertile patients, 161 patients were found to have chronic endometritis with or without endometrial polyps and 156 patients were found to have only endometrial polyps after hysteroscopy (39). It shows that the intrauterine environment is also important for pregnancy. Hysteroscopy is an appropriate technique for accurate uterine evaluation before treatment of infertility. It is the gold standard for the diagnosis and treatment of endometrial lesions. The hysteroscopy appearance of endometrial hyperplasia and cancer is typical and relatively easy to detect (40). Hysteroscopy and endometrial biopsy is recommended to exclude recurrence of endometrial lesions before embryo transfer.

##### 4.1.4.3 Conservative treatment protocols

Options of conservative treatment for endometrial lesions may also affect reproductive outcomes. The current mainstream conservative treatment options are oral progestins, levonorgestrel intrauterine device (LNG-IUD), gonadotrophin releasing hormone analogue (GnRHa), and hysteroscopy. Oral progestins are mostly used (41). However, long-term oral progestins may cause side effects such as weight gain and liver function impairment. Several studies have shown that LNG-IUD combined with oral progesterone or GnRHa caused a higher rate of complete remission (CR) than LNG-IUD monotherapy (42, 43), while hysteroscopy combined with oral progesterone or LNG-IUD also resulted in a higher CR rate (44, 45). The relevant guidelines of the European Society of Human Reproduction and Embryology stated that the combination of oral progesterone and/or LNG-IUD after hysteroscopic tumor resection was the most effective treatment for fertility preservation compared to other treatments (7). A meta-analysis showed that the CR rates in the oral progesterone group, the LNG - IUS combined with GnRHa or progesterone group, and the hysteroscopic electrosurgery combined with progesterone group were 76.3%, 72.9%, and 95.3%, respectively; the recurrence rates were 30.7%, 11.0%, and 14.1%; and the pregnancy rates were 52.1%, 56.0%, and 47.8%, respectively (46). Therefore, combined progestin therapy, LNG-IUS combined with GnRHa or progestin therapy after hysteroscopic electrosurgery can achieve better outcomes for endometrial lesions than progestin therapy alone. But whether it is beneficial for improving patients’ reproductive outcomes needs to be further discussed in higher quality grade studies.

##### 4.1.4.4 Time to CR of endometrial lesions

The impact of the time for CR of endometrial lesions on patients’ reproductive outcomes is controversial. A retrospective study by Yuan Fan et al. (19) showed that patients with a CR time of <6 months were more easily to get pregnant than those with a CR time of >6 months.It suggests that the longer the time of obtaining a CR may have a lower pregnancy rate. However, another retrospective study concluded that CR had no effect on pregnancy outcomes (13). Therefore further studies with multicenter and large sample data are needed.

As to when to initiate ART after CR for endometrial lesion, it remains controversial. Gynecologic oncologists prefer to continue maintenance therapy treatment for at least 2 ~ 3 months to ensure that at least 2 histologically confirmed normal endometriums are obtained, whereas reproductive specialists tend to recommend that patients start ART as early as possible after obtaining a CR (22). The results of a retrospective study by Yaxing Guo et al. (22) showed that a shorter interval between CR and IVF initiation might be a positive factor for live birth. A retrospective study by Du Xiaoguo et al. (20) reported that there was no significant difference in the rates of implantation, miscarriage, and pregnancy between patients with immediate fertilization after complete remission of endometrial lesions and those with fertilization after 3 months, whereas the recurrence rate in the group with fertilization after 3 months was significantly higher than that in the group with immediate fertilization. A retrospective cohort published by Ziyi Song et al. (47) found that initiation of IVF 3 months after CR demonstrated a trend toward an increased number of oocytes, resulting in better controlled ovarian hyperstimulation outcomes for patients. On the other hand, endometrial lesions recurrence is a clear factor affecting pregnancy success (18, 19). Therefore, it is reasonable to encourage patients to undergo IVF as early as possible after obtaining CR to reduce the chances of lesion recurrence and shorten the duration of fertility.

### 4.2 Factors affecting pregnancy outcome: in the process of ART

Patients with AEH/EC can choose the appropriate method of assisted conception based on the appropriate ART indications. The following section focuses on the impact of the IVF-ET process of assisted conception on the patient’s pregnancy outcome.

#### 4.2.1 Controlled ovarian stimulation protocols

Conventional superovulation regimens typically expose patients to high estrogen levels, which may increase the risk of tumor recurrence. In recent years, aromatase inhibitors have been widely used in ovulation induction after conservative treatment of early-stage endometrial cancer. Letrozole is a third-generation aromatase inhibitor that effectively blocks estrogen production without affecting endometrial estrogen receptors (48). Scholars at Cornell Medical College in the United States have recommended an ovarian stimulation regimen of letrozole combined with gonadotropins (49). The protocol makes the peak levels of estrogen during ovulation stimulation close to those of natural cycles. And it also reduces the risk of abnormal endometrial hyperplasia induced by supraphysiologic levels of estrogen during ovulation stimulation. Letrozole combined with gonadotropin regimen reduces levels of estrogen exposure without affecting oocyte quality, fertilization rate or number of embryos obtained (50). This suggests an advantage of letrozole in assisted reproduction in patients with AEH/EC. In contrast, Jiazhou Chen et al. (23) concluded that the PPOS (progestin primed ovarian stimulation) regimen was a feasible and safe ovarian stimulation regimen for patients with AEH/EC and results in a higher rate of quality embryos. Shang Jing et al. (51) suggested that GnRH antagonist had an inhibitory effect on the endometrium, which reduced the effects of the accumulation of high doses drugs used in conservative treatment on the offspring. So they recommended that GnRH agonist regimens are preferred for patients with AEH/EC. Recent advancements in ovarian stimulation protocols, including those focused on the luteal phase and those featuring high progesterone levels, offer more options.for patients with AEH/EC. A study found that ovulation stimulation protocols under high progesterone were effective in improving the outcome of assisted conception in such patients without increasing the risk of disease recurrence (23). The disadvantage of those protocols is the inability to perform fresh embryo transfers during the oocytes retrieval cycle, which prolongs the gestation time. Meanwhile, Amanda J. Adeleye et al. (52) showed that placing LNG-IUS in the uterine cavity during ovulation induction and removing it before embryo transfer did not affect total oocyte yield and mature oocyte yield. It also did not affect clinical pregnancy and live birth rates, and antagonized the effects of estrogen during ovarian stimulation (53).It will decrease the risk of recurrence of endometrial lesions. Due to the small sample size and heterogeneity of current studies on the effects of different ovarian stimulation regimens on assisted conception outcomes in patients with AEH/EC, prospective and multicenter programs are needed to explore the optimal ovulation stimulation regimen for patients with AEH/EC.

#### 4.2.2 Embryo transfer protocols

There are fewer studies on the effect of fresh embryo transfer or frozen-thawed embryo transfer (FET) on the outcome of ART in patients with AEH/EC. A retrospective cohort study which included a total of 259 ET cycles in patients with AEH/EC showed live birth rates were comparable between the two types of embryo transfer, and a significant increase in the incidence of maternal complications and hypertensive syndromes of pregnancy in patients with frozen-thawed embryo transfers (16). This may be due to the presence of exogenous progestogens and estrogens in the artificial cycles (54). However, frozen-thawed embryo transfer reduced the effects of ovulation stimulation on the endometrium and significantly reduced the risk of moderate-to-severe OHSS (55). Therefore, it deserves further exploring the impact of fresh embryo transfer or frozen-thawed embryo transfer on the outcome of assisted conception in patients with AEH/EC. To shorten the time to reach pregnancy, we should transfer fresh embryos,depending on the status of the endometrium during the superovulation cycle. Patients with multiple cycles of overian stimulation need close monitor of endometrial lesions.

There are fewer studies related to FET endometrial preparation regimens on AEH/EC pregnancy outcomes, worse still there is no strong evidence to support that a particular endometrial preparation regimen is superior for pregnancy outcomes. A retrospective study published by Qi Dan et al. (56) showed that the choice of endometrial preparation protocol for frozen embryo transfer did not affect the live birth rate, clinical pregnancy rate, or biochemical pregnancy rate in patients with endometrial hyperplasia. Therefore, the selection of the appropriate endometrial preparation program for frozen embryo transfer should be based on the individual patient’s circumstances. This enables patients to achieve a clinical pregnancy more efficiently. For example, endometrial preparation with natural cycles is recommended for those who need frozen embryo transfer, and endometrial preparation with letrozole-induced ovulation regimen is available for those with combined PCOS or ovulation disorders.

It has not been determined whether the number and quality of embryos transferred in patients with AEH/EC affect pregnancy outcomes differently than in the normal population. A retrospective study which included 179 frozen embryo transfer cycles showed a single embryo transfer rate of 95.5% in patients with endometrial hyperplasia, with no statistically significant correlation between the number of embryos transferred and the rate of live births in the gestational cycle (56). Considering the need for patients with AEH/EC to obtain a pregnancy as soon as possible after the lesion remission, whether the limit on the number of embryos to be transferred should be appropriately liberalized deserves further discussion.

## 5 Summary

Factors such as age, ovarian reserve function, metabolic syndrome, lesion staging and grading, and treatment regimen not only influence endometrial lesions onset, progression, and regression but also affect the patient’s fertility and outcome of assisted conception. The optimal timing, ovarian stimulation regimen and embryo transfer protocols for ART are yet to be further elucidated by higher quality research. In the comprehensive management of patients with AEH/EC, it is of significant clinical importance to reduce the impact of unfavorable factors on fertility and offer appropriate advises.

This review analyzes the research progress in pregnancy outcomes of ART and the influencing factors of ART in AEH/EC patients, and brings hope for patients who still have fertility needs. It offers suggestions for practitioners in choosing suitable solution for such patients. However, there is no consistent evidence on which protocol of ART treatment is the most beneficial for AEH/EC patients after conservative treatment. Robust and high-quality RCTs are needed.

## Author contributions

YJ: Writing – original draft. YL: Writing – review & editing, Funding acquisition. CC: Writing – original draft. YL: Writing – original draft. HX: Writing – original draft. BZ: Writing – review & editing, Funding acquisition.
